# Assessing the Transferability of Peer‐Assisted Ultrasound Training for Medical Students: A Comparative Study Between Two Institutions in Germany and the UK

**DOI:** 10.1002/ca.70098

**Published:** 2026-02-18

**Authors:** Fabian Bauer, Leo F. Nonnenbroich, Leonie Henningsen, Edward Wakefield, Ansh Tandon, Arun J. Thirunavukarasu, Florence Bradshaw, Cecilia Brassett, Ralph A. Nawrotzki

**Affiliations:** ^1^ Institute for Cell Biology and Anatomy Heidelberg University Heidelberg Germany; ^2^ Division of Radiology German Cancer Research Center (DKFZ) Heidelberg Germany; ^3^ Institute for Diagnostic and Interventional Radiology, Faculty of Medicine and University Hospital Cologne University of Cologne Cologne Germany; ^4^ Human Anatomy Centre, Department of Physiology, Development and Neuroscience University of Cambridge Cambridge UK; ^5^ School of Clinical Medicine University of Cambridge Cambridge UK; ^6^ Nuffield Department of Clinical Neurosciences, Medical Sciences Division University of Oxford Oxford UK; ^7^ Faculty of Medicine Heidelberg University Heidelberg Germany

**Keywords:** peer‐assisted‐learning, SASH, transferability, ultrasound

## Abstract

Ultrasound (US) is a clinically important imaging modality that can also enhance medical students' understanding of anatomy, physiology, and pathology. However, its integration into preclinical curricula often remains limited due to challenges such as resource constraints and instructor availability. To address these shortcomings, we implemented and evaluated a peer‐assisted learning (PAL)‐based US course—Summer School of Anatomy‐based Sonography Heidelberg (SASH)—with a daughter course at a second institution, the University of Cambridge (Cam‐SASH). Both programs focused on teaching fundamental US techniques through a structured, tutor‐led curriculum including an accompanying course manual. In 2022, we evaluated both programs prospectively, including 36 medical students. Over 1 week, student tutors trained participants in B‐mode abdominal US through lectures, hands‐on practice, and assessments, including Objective Structured Clinical Examinations (OSCEs) and pre‐ and post‐course multiple‐choice tests of anatomical knowledge. Post‐course knowledge levels were comparable between Hei‐SASH and Cam‐SASH participants, with no significant differences observed in multiple‐choice tests or OSCE performance (*p* ≥ 0.17). Feedback was overwhelmingly positive, with students reporting increased confidence and proficiency in performing US scans and interpreting images. This study highlights the transferability of PAL‐led US courses, with comparable outcomes between institutions. Our findings support the inclusion of such programs in undergraduate medical curricula, as they provide a cost‐effective and scalable solution to resource limitations. By enabling students to gain hands‐on experience with real‐time imaging, these courses bridge the gap between theoretical learning and clinical application, equipping future physicians with essential diagnostic skills.

## 1 Introduction

In anatomy education, state‐of‐the‐art illustrations have long helped students understand the human body. Experiential learning, such as involvement in research projects, can enhance students' appreciation of anatomy (Sinha et al. 2025). Recent advancements have involved integrating ultrasound (US) into the study of anatomy (Patel et al. 2017; Royer and Buenting Gritton 2020). Using US, students can obtain immersive, real‐time 2D images of structures, compare anatomical similarities and differences among individuals, and study the shapes and topographies of structures under physiological and pathological conditions. In other words, US provides a unique, live, and in vivo perspective into the human body (So et al. 2017). We propose that US training should be more widely integrated into teaching anatomy, provided that sufficient resources are available, and students value the opportunity to learn US early in their medical education.

Studies have consistently shown that medical students value the chance to develop US skills within the context of their anatomy classes. For example, researchers have employed pre‐ and post‐course surveys to assess changes and improvements throughout the instructional period. They found that students reported perceived enhancements in performing US scans, interpreting US images, and their perceived anatomical knowledge level (Brown et al. 2012; Dreher et al. 2014; Edwards et al. 2023; Ivanusic et al. 2010; Lufler et al. 2022; Royer et al. 2017; Swamy and Searle 2012; Sweetman et al. 2013). Students also articulated the value of early exposure to US and the proficiency to conduct US scans as being advantageous for future clinical encounters. While there is evidence indicating that learning using US can improve students' comprehension of anatomy (Knudsen et al. 2018; Royer et al. 2017; Tshibwabwa and Groves 2005), other studies suggest that traditional teaching methods such as cadaver‐based instruction (Griksaitis et al. 2012), seminars (Knudsen et al. 2018), or narrated videos (Vandenbossche et al. 2023) are equally effective in teaching the subject. Given these reports, it is reasonable to question the value of investing time and resources in implementing preclinical US curricula (Feilchenfeld et al. 2017; Solomon and Saldana 2014). Furthermore, many institutions may hesitate to incorporate preclinical US teaching due to insufficient specialist knowledge. Possessing expertise in anatomy does not necessarily equip educators with the skills required to interpret US images.

Once institutions have decided to implement a preclinical US curriculum, administrators often face challenges with scheduling, infrastructure, and personnel resources (Bahner et al. 2014; Tandon et al. 2024). There are different views on the optimal timing and structure for US curricula. For instance, some have achieved success by aligning their courses with the dissection class (Allsop et al. 2021; Lufler et al. 2022; Royer et al. 2017), while others have implemented longitudinal US curricula spanning over four years of training (Hoppmann et al. 2011). Meanwhile, our institutions and others have opted to deliver US training in condensed, coherent blocks of topics (Khoury et al. 2019; Kloth et al. 2022). An additional barrier for curriculum planners is ensuring participants have sufficient access to US equipment. Portable US devices are expensive, occupy space, and practice time is limited. If these parameters pose limitations, one might consider using more affordable, yet effective, handheld US devices (Edwards et al. 2023; Ireson et al. 2019; Swamy and Searle 2012). These devices offer flexibility and accessibility while delivering the desired educational content.

Recruiting and retaining high‐quality instructors for US education presents the biggest obstacle. In the UK, US training is typically delivered by experienced clinicians and academic staff (McCormick et al. 2023). However, the limited availability of clinicians can pose a challenge, especially with the growing demand for qualified sonographers in the UK workforce (Waring et al. 2018). One potential solution to this challenge is to explore the option of training medical students to serve as US tutors in peer‐assisted learning (PAL) (Brierley et al. 2022; Burgess et al. 2014). Several randomized controlled trials have demonstrated the effectiveness of PAL (Celebi et al. 2012; Hari et al. 2023) and that PAL is effective for long‐term knowledge retention of US knowledge in medical students (Nourkami‐Tutdibi et al. 2020). These and further studies (Ahn et al. 2014; Dickerson et al. 2017; Oberoi et al. 2021) suggest that PAL‐based US courses effectively enhance students' US knowledge and skills, and are not inferior in their outcome compared to courses taught by clinical staff.

At Heidelberg University, PAL was introduced into the US‐based anatomy curriculum 20 years ago. Between 2010 and 2024, 280 students have been trained as tutors, which enabled us to recruit 40 tutors per year since 2010 to teach 350 first‐year medical students in a five‐day course on abdominal US. In 2016, building on the success of this training program, we selected tutors to teach a group of international students in abdominal US. This was subsequently named the Summer School of Anatomy‐based Sonography Heidelberg (SASH). SASH is based on our institution's preclinical US program and includes extensive hands‐on sessions in the teaching hospitals of Heidelberg University. Over the years, our team of tutors and former SASH participants have worked together to establish similar summer schools at their respective institutions. For example, in 2022, we launched Cam‐SASH at the University of Cambridge. Cam‐SASH follows the same PAL‐based course structure and learning methods as Hei‐SASH at Heidelberg University.

In this report, we hypothesize that we could adapt our training model to work within a different setting, as Heidelberg US tutors have successfully trained medical students and international SASH participants at our institution. Given similar institutional support at the University of Cambridge, along with existing resources and a comparable Cam‐SASH cohort, we predicted that both Hei‐SASH and Cam‐SASH tutors could also provide US training for medical students at Cambridge. We defined a successful transfer of our training model as the achievement of comparable outcomes in anatomy and Objective Structured Clinical Examination (OSCE) tests by participants of Hei‐SASH and Cam‐SASH, along with similar ratings in feedback surveys. In this work, we describe in detail the implementation of the SASH program at two institutions. We believe that our bi‐institutional, bi‐national, and PAL‐led approach to US training for medical students is unique, and that the insights gleaned from both programs will be of interest to medical educators who are seeking to integrate US into their anatomy curricula.

## 2 Materials and Methods

### 2.1 Study Design

This prospective, review board‐approved (S‐441/2022, University of Heidelberg) pilot, descriptive study investigated the impact of SASH on medical students' US knowledge and addressed the transferability of SASH from one institution to another. The study cohort comprised a convenience sample of all students enrolled in the two summer schools in 2022. The number of participants was constrained by the fixed capacity of the small‐group, hands‐on courses at both institutions, and a formal a priori power calculation was therefore not performed. The study was intentionally designed without a traditional‐instruction or no‐intervention control group, as our primary aim was not to evaluate PAL against alternative teaching formats but to explore whether an existing PAL‐based curriculum could be transferred to another institution with comparable outcomes.

The (self‐)evaluations and examinations took place contemporaneously during each course. Test scores were compared to assess any differences in learning outcome and transferability. Additionally, we documented our reflections and assessment of challenges during the course implementation based on feedback received during or at the end of each SASH course since 2016. Our reflections excluded the year 2020 when US teaching could not take place due to COVID‐19 control measures.

### 2.2 Study Cohort

Between 31 July and 13 August 13, 2022, all participants in Hei‐SASH and Cam‐SASH were asked to participate in this study. The inclusion criteria comprised written informed consent, being at least 18 years of age, and full participation in the Hei‐SASH or Cam‐SASH courses.

Applicants who wanted to participate in the Hei‐SASH 2022 summer school had to meet specific criteria. These included being a medical student at an accredited international medical school and passing anatomy exams at their home institution. Participants also had to demonstrate proficiency in English, submit letters of recommendation from senior university staff or previous SASH participants, provide a curriculum vitae, and write a motivational statement. Applicants for Cam‐SASH were asked to submit a personal statement, curriculum vitae, and a statement from a member of staff from their faculty. Participants were chosen based on interest in US, educational background, relevant work experience, and understanding of US relevance. Application review was blinded. Based on ranking by the senior student tutors, the top 20 and 16 applicants were admitted to Hei‐SASH and Cam‐SASH in 2022, respectively. Participants had to provide written informed consent for study inclusion. The study adhered to ethical guidelines, ensuring participant confidentiality and voluntary participation prior to data collection.

### 2.3 Train the Tutor Program

Both Hei‐SASH and Cam‐SASH summer schools were taught by 3rd, 4th, 5th, and 6th‐year medical students, who had been carefully selected and trained by Heidelberg University's train‐the‐tutor (TTT) program. The TTT program, led by senior tutors and the study's lead author, selected 20 prospective tutors annually based on their performance in the intramural US course, teamwork abilities, and teaching skills. These potential tutors then underwent training over four weekends in teaching small groups, management of challenging participants, and ensuring the achievement of all the goals listed in the course manual. As part of the TTT program, tutors received explicit training in the use of standardized checklists for pretests and OSCE assessments, including guidance on consistent scoring, structured feedback delivery, and minimizing examiner‐related variability. The program also involved a 2‐week rotation at an US unit, which could be in a teaching hospital or private practice. It ended with a yearly team‐building retreat that also provided an opportunity to discuss upcoming changes to the US course. The rationale of the TTT program is explained in Appendix S1.

### 2.4 Course Content and Structure

Hei‐SASH and Cam‐SASH have identical curricula. SASH provides a one‐week introductory course in B‐mode abdominal US designed for beginners, including 30 h of intensive instruction. The course comes with a comprehensive manual that covers the fundamental principles of ultrasonography, including the physical properties of sound waves and essential concepts such as absorption, reflection, and artifacts. The manual explains the best probe‐handling techniques for each organ to achieve optimal scanning outcomes. It also provides guidance on distinguishing between pathological and physiological findings in ultrasonographic imaging.

**FIGURE 1:**
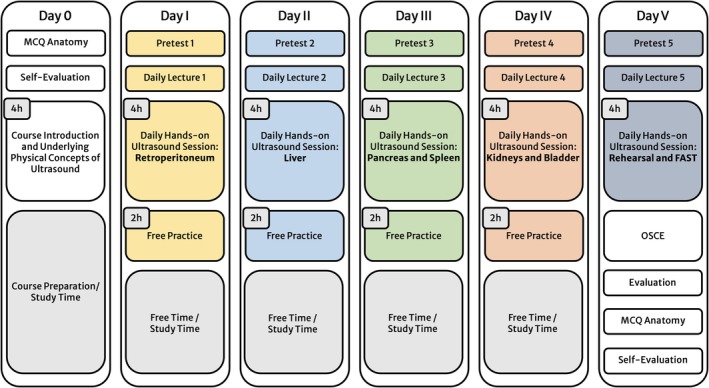
Course concept and data collection. FAST, focused assessment with sonography in trauma, MCQ, multiple‐choice question; OSCE, objective structured clinical examination.

SASH aims to aid students in the real‐time identification of anatomical patterns in 2D images. As an integral aspect of the course, participants are required to acquaint themselves with and commit to memory the anatomical topography across 12 standard planes in the abdomen. These planes represent the most scrutinized areas in abdominal US (Hofer et al. 2012, 2000; Teichgräber et al. 1996). The introductory session covers the fundamental principles of US and its background physics. Subsequently, the course is divided into five segments, each lasting approximately 4 h, with the whole program spanning 5 days. Each segment is comprehensively described in the course manual, delineating the retroperitoneum (Day I), liver and gallbladder (Day II), pancreas and spleen (Day III), kidneys and urinary bladder (Day IV), and Focused Assessment with Sonography in Trauma (FAST; Day V). Each day encompasses an interactive discussion of specific pathologies pertinent to the respective topic. To provide thorough hands‐on training, instruction is conducted in small groups of no more than five participants, with each group having access to one US device and supervised by a student tutor. Two hours daily are also set aside for free practice sessions, allowing for review and supplementary practical exercises. Figure 1 depicts an overview of the course design.

Before starting each course segment, all participants were required to take a pretest. These tests consisted of written questions and the task of drawing one of the 12 standard planes from memory. The questions and standard planes were selected randomly and covered upcoming as well as previous course material. For each pretest, a maximum of eight points was awarded for drawing the standard plane, with two additional points for answering the theoretical questions. This approach was meant to ensure that participants had the necessary theoretical and US topographical knowledge to ensure sufficient time for hands‐on US training. At the end of the SASH program, participants had to complete a practical OSCE. Participants rotated through three parts during this assessment, with 8 min per station. Each OSCE rotation comprised a practical task (20/30 points) and answering two to three oral questions (10/30 points). The practical components of the initial two rotations involved the completion of a randomly assigned US task from the SASH program. In the third rotation, all participants were required to perform a Focused Assessment with Sonography for Trauma (FAST) examination as part of the practical assessment. All OSCE stations were accompanied by predefined checklists with fixed scoring criteria to promote standardized assessment across examiners. Appendices S1 and S3 provide an example pretest and OSCE rotation evaluation form.

The Cam‐SASH curriculum was first implemented at the University of Cambridge in 2022. Cam‐SASH followed the same structure and fundamental principles of the SASH program, developed at Heidelberg University. Prior to the establishment of Cam‐SASH, tutors of Heidelberg University had provided training for Cambridge medical students and those who were later to become Cam‐SASH tutors. Through several visits to each other's institutions, both tutor teams collaborated to develop comparable curricula. The senior authors of this publication (RAN and CB) oversaw the implementation of Hei‐SASH and Cam‐SASH.

### 2.5 Data Collection and Evaluation

We conducted two sets of multiple‐choice question (MCQ) tests to assess the participants' anatomical knowledge before and after the SASH program. The initial MCQ design was conducted by the corresponding author (RAN) in collaboration with five advanced US tutors and anatomy staff. From a larger pool of 120 items, 60 MCQs were selected based on their higher difficulty and discrimination indices, as determined from the responses of a cohort of 297 students (Appendix S4). Each MCQ test contained 30 questions in a best‐of‐three format, focusing on anatomical topography, including US, MR, CT, and angiography images. An additional response option, “don't know,” was included to discourage guessing. Appendix S5 shows three MCQ examples. All participants completed self‐evaluation forms before and after the SASH course to evaluate their perceived improvement in anatomical knowledge. Participants were asked to assess their understanding of anatomical topography, pathologies, and overall proficiency in ultrasonography using a six‐point Likert scale (1 = excellent, 2 = very good, 3 = good, 4 = poor, 5 = very poor, 6 = nonexistent). In addition, participants were asked to assess the course concept. They utilized a symmetrical five‐point Likert scale to evaluate Hei‐SASH and Cam‐SASH (1 = completely agree, 2 = agree, 3 = neutral, 4 = disagree, 5 = completely disagree). Figure **1** depicts the timeline for the data collection. All data were anonymized by assigning participant numbers.

### 2.6 Statistical Analysis

Descriptive statistics are presented as the mean accompanied by the standard deviation (SD) for normally distributed quantitative variables and as the median accompanied by interquartile ranges (IQR) or ranges for quantitative variables that were not normally distributed. The differences in MCQ, OSCE, and pretest scores between Hei‐SASH and Cam‐SASH were assessed using the Mann–Whitney *U*‐test. Evaluations were analyzed descriptively, considering their subjective nature. Data analysis and visualization were performed using R version 4.0.3 (R Foundation for Statistical Computing, Vienna, Austria).

## 3 Results

### 3.1 Study Cohort

Between 2016 and 2022, 101 international students participated in the SASH program. In 2022, the program enrolled 36 medical students from 11 different universities. These students participated in either the Hei‐SASH (20 students, 13 males, and seven females) or Cam‐SASH (16 students, seven males, and nine females) summer school. Participants in Cam‐SASH or Hei‐SASH gave written consent and actively participated in all aspects of the study. Participants' characteristics are displayed in Table 1.

**TABLE 1:** Characteristics of the Hei‐SASH and Cam‐SASH course participants in 2022.

	Hei‐SASH	Cam‐SASH	Total
Participants (*n*)	20	16	36
Age (median; range)	23; 19–30	21; 19–22	21; 19–30
Sex (M:F)	13:7	7:9	20:16
Sending institutions (*n*)	10	1	10
Year of studies (median; range)	4; 1–6	3; 3–4	3; 1–6
Prior time spent on learning ultrasound			
None (*n*)	12	4	16
≤ 10 h (*n*)	5	9	14
10 to < 20 h (*n*)	3	2	5
20 to < 30 h (*n*)	0	0	0
30 to < 40 h (*n*)	0	0	0
40 h to < 50 h (*n*)	0	0	0
≥ 50 h (*n*)	0	1	1

The majority of the study participants were affiliated with the University of Cambridge (center 1; *n* = 17), followed by Charles University Prague (center 2; *n* = 5) and the University of Milan (center 3; *n* = 5). Before participating in Hei‐SASH or Cam‐SASH, most participants had received less than 11 h of US training. Only 25% of all study participants had access to US training at their home institution. An additional 28% of participants had the option to arrange US practice independently. Lastly, 47% of participants did not have the opportunity to acquire US skills at their home institution.

#### 3.1.1 Self‐Evaluation Through Hei‐SASH and Cam‐SASH

**FIGURE 2:**
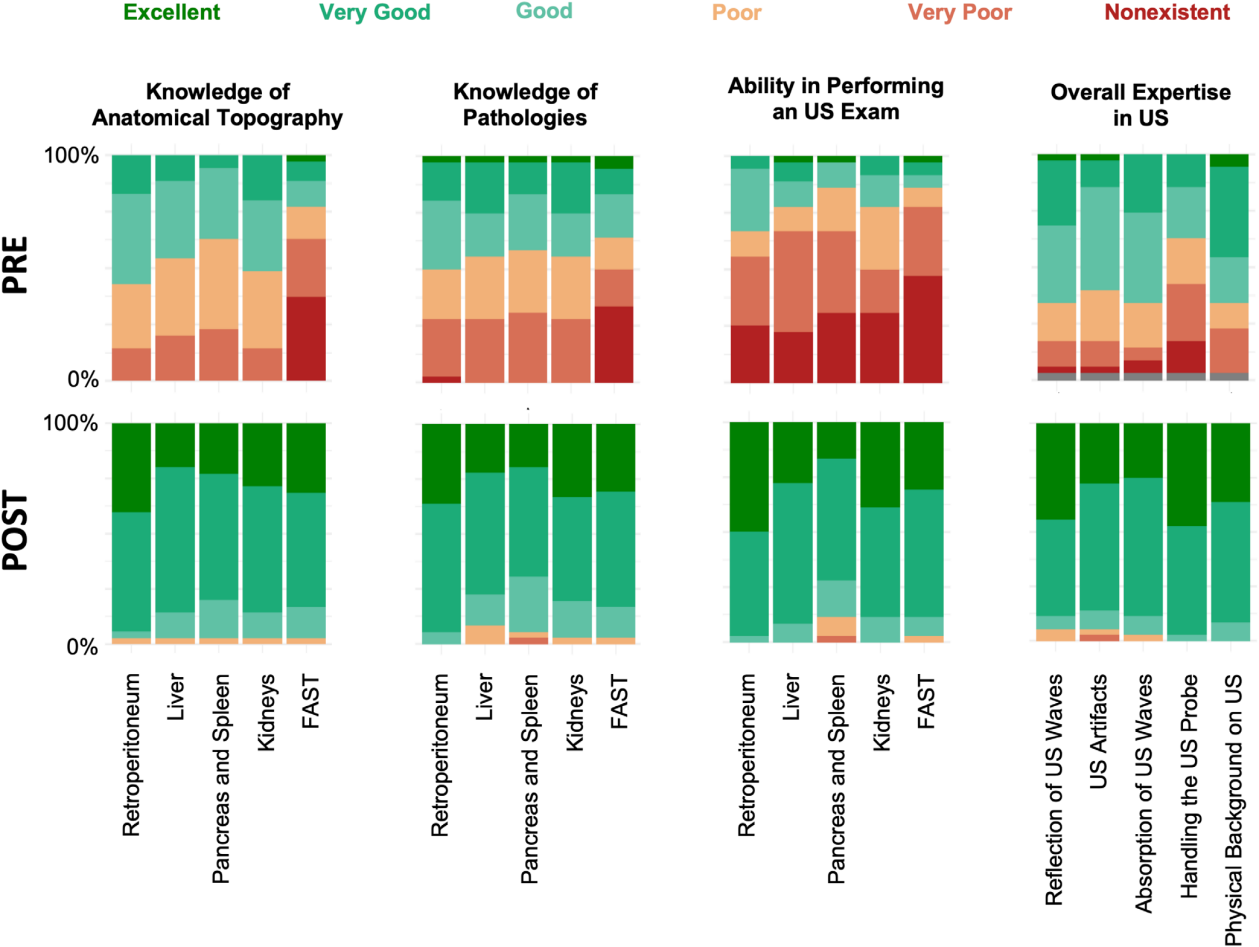
Likert‐scale plots representing self‐evaluation answers prior (PRE) and after (POST) course. Results are shown relatively scaled for Hei‐SASH and Cam‐SASH participants combined. FAST, focused assessment with sonography in trauma; US, ultrasound.

To evaluate perceived course benefit, participants were asked to complete a self‐evaluation form before and after the course. Across all categories, participants indicated an increase in their confidence levels, with the most pronounced subjective enhancements observed in their anatomical and pathological knowledge as well as their ability to conduct a FAST examination (Figure 2).

#### 3.1.2 Impact of the Course on Anatomical Knowledge

**FIGURE 3:**
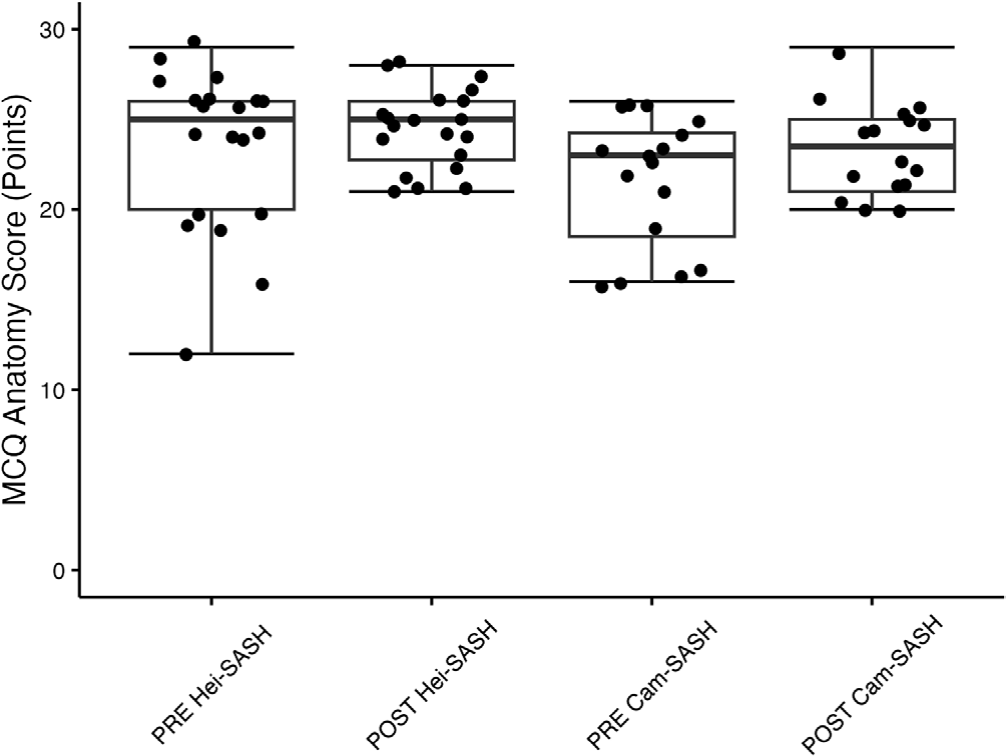
Multiple‐choice questionnaire (MCQ) results before (PRE) and after (POST) attending the course. Box plots display the median, first quartile (Q1), and third quartile (Q3) with whiskers representing 1.5 times the interquartile range.

The potential impact of the course on anatomical knowledge was evaluated through the MCQ tests before and after the course. Due to the ambiguous phrasing of two questions, the maximum test score for each test was 29.0 points due to the exclusion of one question from each test. Prior to the course, the median anatomical knowledge score was 25.0 points (86%, IQR: 20.0–26.0) for the Hei‐SASH group and 23.0 points (79%, IQR: 18.5–24.3) for the Cam‐SASH group (*p* = 0.07). Following the completion of the course, the median anatomical knowledge score was 25.0 points (86%, IQR: 22.8–26.0) for the Hei‐SASH group and 23.5 points (81%, IQR: 21.0–25.0) for the Cam‐SASH group (*p* = 0.17). There were no statistically significant differences in the absolute improvement between both groups (*p* = 0.52). Figure 3 shows the detailed MCQ results for Hei‐SASH and Cam‐SASH.

#### 3.1.3 Pretest and OSCE Results

In the pretests, the median score in the Hei‐SASH group was 7.3 points (73%, IQR: 5.5–8.4), while it was 6.2 points (62%, IQR: 4.8–8.3, *p* = 0.94) for the Cam‐SASH group. It is important to note that the pretests were specifically designed to gage participants' preparedness for the course and may not fully reflect the actual learning outcomes.

**FIGURE 4:**
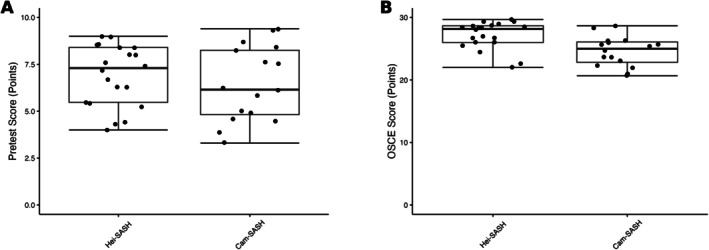
Pretest (A) and OSCE scores (B) in Cam‐SASH and Hei‐SASH. Box plots display the median, first quartile (Q1), and third quartile (Q3) with whiskers representing 1.5 times the interquartile range.

In the OSCEs, the median score was 28.2 points (94%, IQR: 26.0–28.7) in the Hei‐SASH group and 25.0 points (83%, IQR: 22.8–26.1) in the Cam‐SASH group (*p* = 0.79). Figure 4 shows the details of the pretest and OSCE scores.

#### 3.1.4 Course Evaluations

**FIGURE 5:**
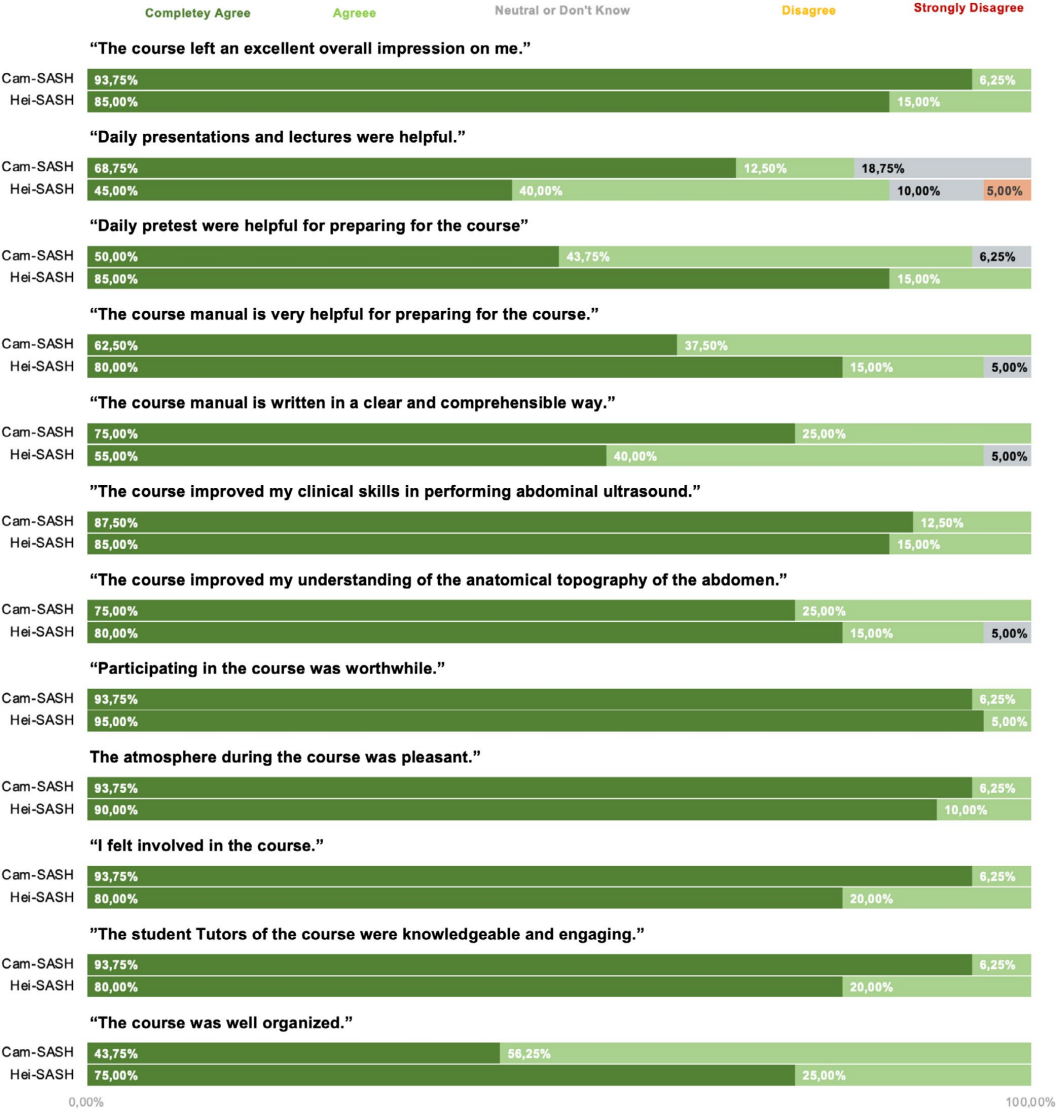
Bar chart encoding the mean values of the evaluations based on a Likert score with 1 = completely agree and 5 = completely disagree. Cam‐SASH in blue, SASH in red.

All participants expressed a strong inclination to recommend the course to their colleagues. Figure 5 displays the results of the course evaluation based on a 5‐point Likert scale.

## 4 Discussion

This report contributes to the discourse on the transferability of PAL‐based US curricula in teaching anatomy. We introduce the SASH program, a week‐long, hands‐on abdominal US course designed for international medical students, which has been implemented at two institutions across two countries (Germany and UK). Our findings show that well‐trained student tutors could teach abdominal US at both institutions with a comparable level of effectiveness, as supported by the participants' content‐specific knowledge, their proficiency in performing scans, and their heightened confidence and enthusiastic self‐assessments after completing the course. The comparable results between both courses suggest that PAL‐led US courses for medical students can be transferred and implemented at external institutions.

Previous studies on PAL‐led US have examined the generalizability of their findings. For example, three studies randomly assigned their volunteer students to experimental and control groups. Through these trials, researchers have provided strong evidence that PAL‐led US teaching can produce equal (Celebi et al. 2012) or even better outcomes than staff‐based instruction (Hari et al. 2023; Nourkami‐Tutdibi et al. 2020). However, these experimental and other studies on PAL‐led US courses (Ahn et al. 2014; Dickerson et al. 2017; Oberoi et al. 2021) did not select their sample randomly from the entire cohort of students at their institutions; rather, the participants were motivated volunteers instead. There is only one randomized controlled trial in the literature where almost all the students from one academic year were randomly assigned to study course content using either US instruction or a traditional cadaver‐based intervention (Griksaitis et al. 2012). This report found that both modes of instruction were equally effective in teaching the subject matter. It is therefore still unclear whether all the students from one's institution will benefit from US training. Moreover, anatomists who worry about the high cost and somewhat unclear effectiveness of using US in their teaching might consider other factors to indicate that teaching US to medical students is a good investment.

A key factor of US training for medical students is their increased confidence in performing an essential clinical procedure. For instance, our survey data, which align well with observations from previous studies (Dreher et al. 2014; Lufler et al. 2020; Patel et al. 2017; Patten 2015; Royer et al. 2017; Swamy and Searle 2012; Sweetman et al. 2013), indicate that participants in both Hei‐SASH and Cam‐SASH experienced a marked improvement in their perceived US competencies. It would appear to us that, similar to the multiple advantages of performing dissections in gross anatomy (Flack and Nicholson 2018; Korf et al. 2008; Rizzolo and Stewart 2006), the benefits of teaching US to medical students are diverse (e.g., enhanced understanding of anatomy, understanding the clinical relevance of anatomy, improved pattern recognition skills or proficiency in operating the US device) and may differ in their impact from one student to another. Overall, we believe that teaching US in preclinical education can help bridge the theory‐practice gap many students encounter in their medical training.

The SASH program offers a week‐long curriculum in US at Heidelberg University and the University of Cambridge for medical students worldwide. Our study included all participants of the SASH program, such that the assessments are based on a convenience sample and cannot be easily generalized to a larger population. When random sampling is not feasible, replication is essential to increase confidence in how well the results apply to various populations or settings (Fraenkel et al. 2022). Our research has replicated findings from other studies; our study is similar and has yielded comparable results regarding the effectiveness and acceptance of US training in teaching anatomy (Dreher et al. 2014; Ivanusic et al. 2010; Khoury et al. 2019; Kloth et al. 2022; Knudsen et al. 2018; Lufler et al. 2022; Nelson et al. 2017; Patel et al. 2017; Royer et al. 2017; Swamy and Searle 2012; Tshibwabwa and Groves 2005). We found that the SASH courses enhanced participants' perceived and actual knowledge of real‐life anatomy. After completing their courses, participants also reported increased confidence in using US equipment. Additionally, our findings align with the conclusions from comprehensive literature reviews in the field (Birrane et al. 2018; McCormick et al. 2023; So et al. 2017).

Our report extends previous studies by exploring the transferability of our local US course (Hei‐SASH) to an institution in another country (Cam‐SASH). We found no significant differences in participants' performances in anatomy MCQs or OSCEs between those enrolled in Hei‐SASH or Cam‐SASH. Additionally, the courses received outstanding ratings at both institutions. While it may not be surprising that a well‐tested curriculum such as SASH can be transferred from one institution to another, having near‐peer tutors teach US without the assistance of clinical staff does not accord with expert recommendation. The European Federation of Societies for US in Medicine and Biology (EFSUMB) indicated that preclinical US curricula are “probably best organized and implemented by a multidisciplinary group including internal medicine, radiology, and experienced US specialists with the course directors of the anatomy, physiology, and pathophysiology preclinical courses” (Cantisani et al. 2016). We acknowledge this view and agree that receiving instruction from experienced specialists teaching US would be ideal. However, our data suggest that student tutors can effectively teach anatomical US, thus allowing students to observe functional anatomy in real time and help bridge the gap between theoretical knowledge and practical application, and identify structures, relationships, and spaces in the living body. The SASH curriculum has consistently helped us achieve that goal, first in Heidelberg and now in Cambridge, by adhering to anatomy‐based scanning objectives, supplemented with pathological US features as needed. We encourage fellow anatomists to use near‐peer tutors for US instruction in appropriate regions of the human body.

Differences in the evaluation of individual course components between Hei‐SASH and Cam‐SASH participants may reflect contextual or cohort‐specific factors rather than differences in the underlying curriculum. These include individual learning preferences, prior educational experiences, and expectations shaped by local teaching cultures. Given the pilot nature of this study, we did not systematically assess cultural influences on learning behavior. However, identifying such differences in course evaluations was an intended part of the implementation process and served as a valuable tool for course refinement.

There are limitations to our study. First, the overall sample size was modest, and our cohort comprised motivated and well‐prepared medical students who represent only a small and pre‐selected fraction of all medical students. The limited number of participants, together with the convenience sampling approach prevents adjustment for potential confounders. This is a direct consequence of the organizational constraints of the SASH summer schools, which are designed as intensive, small‐group, hands‐on courses with limited capacity. Our primary aim was therefore to explore whether our established PAL‐based US curriculum could be transferred to another institution with comparable learner outcomes. The unavoidable selection bias may help explain why, upon analyzing the outcomes of the anatomy MCQs, we found that the mean anatomy knowledge of participants from Hei‐SASH and Cam‐SASH only slightly improved upon completing the course. We observed, however, a convergence‐toward‐mastery effect, as the post‐test scores showed less variability than the pre‐test scores. This effect might indicate that participants with limited prior anatomy knowledge benefited from SASH's instructional support and detailed feedback, despite only limited opportunities for deliberate practice (Ericsson 2015). With their new knowledge and skills, all SASH participants were prepared to continue voluntary practice at their home institutions and further develop expertise in abdominal US. Further research with larger cohorts is needed to determine how individual students benefit from SASH. These studies could offer deeper insights into the program's effectiveness and guide future curriculum development to maximize educational outcomes for diverse learners.

Second, we did not include a traditional‐instruction or no‐intervention control group. As a result, we cannot attribute observed improvements solely to the PAL approach. Our goal, however, was to document the transferability of an established curriculum rather than to isolate the learning effect of PAL. Prior randomized controlled trials have already examined PAL versus clinician‐ or staff‐led US teaching (Celebi et al. 2012; Hari et al. 2023), and our study builds on this work by assessing whether a successful PAL program can be implemented effectively in another educational setting.

Additionally, we did not formally assess inter‐rater reliability for the OSCE evaluations, nor did we conduct examiner calibration sessions beyond the training embedded in our established TTT program. While standardized checklists and tutor preparation were intended to reduce variability, the absence of quantitative reliability data means that inconsistency between examiners cannot be excluded.

Another limitation of our study is that we did not assess students' long‐term retention of knowledge and skills—an essential benchmark of educational effectiveness. While innovative teaching approaches, such as team‐based learning (Emke et al. 2016), simulation training (Offiah et al. 2019), and game‐based teaching (Yu et al. 2025), have demonstrated substantial initial improvements in knowledge, research consistently shows that these early gains tend to decrease over time unless reinforced. We anticipate that SASH will follow a similar pattern. In fact, sustained knowledge and skill retention depend on several key factors, including regular formative assessment (Larsen et al. 2008), prompt and constructive feedback (Hoefer et al. 2017), and ongoing, deliberate practice (Wang et al. 2025). By introducing US through SASH, we lay the groundwork for students to develop a lasting understanding of essential clinical anatomy. Subsequent clinical US curricula further strengthen these skills by offering continued opportunities for hands‐on practice, regular feedback, and targeted assessment. We are confident that students who complete SASH or similar early US curricula enter clinical training with greater confidence and preparedness than students who lack the practical confidence and integration skills fostered by earlier exposure.

It is important to recognize that alternative teaching formats can be just as effective as US courses for teaching applied abdominal anatomy (Griksaitis et al. 2012; Knudsen et al. 2018; Vandenbossche et al. 2023). Indeed, we argue that no single teaching method is universally superior for imparting applied clinical anatomy. Rather, the most successful curricula are those that prioritize clinical relevance, actively engage students through multimodal learning strategies, and provide ample opportunities for hands‐on practice (Estai and Bunt 2016). Moreover, there is increasing recognition that anatomical knowledge and skills must be meaningfully integrated with the demands of clinical practice (Orsbon et al. 2014), and some anatomists argue that knowledge of diagnostic imaging is paramount for teaching clinically relevant anatomy (Leveritt et al. 2016). What, then, are the benefits of integrating US courses such as SASH into preclinical curricula? While such courses alone may not ensure long‐term knowledge retention, they enable medical students to overcome the initial challenges of US practice and initiate continuous skill development early in their training. Additionally, students are encouraged to focus on clinically relevant structures and topographies rather than solely memorizing anatomical content. When combined with other innovative teaching tools, SASH can provide unique insights into the human body and clinical skills that are not accessible through traditional instructional methods.

In future research, efforts could be made to create a database that collects and standardizes data on the effectiveness of their anatomy‐based US curriculum from various US course providers. Two possible models include the freely accessible platforms by the London‐based Educational Endowment Foundation (EEF) (Educational Endowment Foundation 2026) or by the U.S. Department of Education (Institute of Education Sciences of the U.S. Department of Education n.d.). Both platforms offer information on educational interventions that have been shown to be effective. In anatomy education, a similar platform might include data about the short‐term and long‐term effectiveness of an US course, along with additional information to help researchers design and interpret educational studies. For example, it would be interesting to explore the extent to which students benefit from taking US classes. Will weaker students, through US instruction, be able to reach the same levels of understanding and proficiency as their more able and accomplished peers? And can US teaching provide a scaffold for students with weaker spatial recognition skills? Both early research (Rochford 1985) and recent analyses (Roach et al. 2021) consistently demonstrate that students with spatial learning disabilities underperform in traditional cadaver‐based anatomy curricula. Participation in anatomical US courses may improve confidence and pattern recognition among these students, particularly those who struggle to interpret US images.

## 5 Conclusion

This study describes the successful implementation of Hei‐SASH and Cam‐SASH. Moreover, by offering this PAL‐led US course to international students at two institutions in two countries, we show that our course design, which involves near‐peer tutors, is transferable from one educational setting to another. It is hoped that these findings will enable anatomists and curriculum administrators to feel more confident in the recruitment of near‐peer tutors to provide US instruction within a preclinical anatomy curriculum. If the circumstances are right, participants can benefit from US training, and those with poor spatial recognition skills may benefit even more than others.

## Funding

This study was supported by the 4EU+ European University Alliance (2021/F1/06), Ruprecht‐Karls‐Universität Heidelberg, Germany, and the German Academic Exchange Service.

## Ethics Statement

This prospective study was approved by the Institutional Review Board. Informed consent was given by all study participants. The authors attest that this report contains no personal information that could lead to the identification of the participants.

## Supporting information


**Data S1:** Supporting information.

## Data Availability

The data that support the findings of this study are available on request from the corresponding author. The data are not publicly available due to privacy or ethical restrictions.
