# Supplementary material for: Assessing the Transferability of Peer‐Assisted Ultrasound Training for Medical Students: A Comparative Study Between Two Institutions in Germany and the UK
Source: Clin Anat. 2026 Feb 18;39(4):541–52. doi: 10.1002/ca.70098 (PMC13060010; doi:10.1002/ca.70098)

**Assessing the Transferability of Peer-assisted Ultrasound Training for Medical Students: A Comparative Study Between Two Institutions in Germany and the UK**

**Appendix S1**

***Rational of the Train the Tutor program***

The Train the Tutor (TTT) program at the Heidelberg University is a unique initiative, designed to train student ultrasound tutors in communicating learning content, following the peer-assisted learning (PAL) concept. The program's innovative approach involves training prospective tutors under the guidance of experienced student tutors, who in turn are supervised by senior tutors.

The selection process for tutors in the TTT program is rigorous. Prospective tutors are evaluated based on their performance in the pre-clinical US course, their US skills, and their social participation in small groups. Their achievements in an additional mock OSCE exams are also taken into consideration. This thorough evaluation process ensures that only the most qualified candidates proceed to the official selection procedure, which takes place after the students' first state examination at the end of their pre-clinical studies (third year students).

Prospective tutors are invited to attend a selection day where they are required to give a 10-minute presentation on an ultrasound topic, perform ultrasound examinations as part of the mock OSCE, and explain why they are motivated to become future tutors. Following this, the strongest 20 candidates are selected each year and invited to participate in the TTT workshops that take place on two consecutive weekends. During these workshops, small groups of five prospective tutors are guided and supervised by experienced and supervising tutors in regular course sessions. Each candidate receives immediate and exacting feedback by senior tutors on their sonography skills, verbalization of probe handling, and other related skills. This feedback is given through rounds of ultrasound examination by the prospective tutors while being observed by the other members of the group and two senior tutors.

Prospective tutors are evaluated in various ways, including a video recording of their performance during a small ultrasound session. The video is later shown to the prospective tutor, and feedback and suggestions for improvement are provided. In addition, the prospective tutors participate in so-called “trouble-shooting sessions” where they interact with difficult course participants, such as those who are unprepared, overzealous, or disruptive. These sessions are designed to help the tutors learn how to stay focused on the task at hand and maintain a positive working environment for the small group. Prospective Tutors also receive a detailed session on how to test and mark OSCEs and pretests.

After the first two weekends are completed, prospective tutors are scheduled to participate in a workshop retreat that aims to enhance team building, fine-tune any technical skills, and discuss the details and procedures of the upcoming ultrasound course. This retreat is also where the course lead (RAN) of this publication meets to discuss any changes to the course curriculum. All activities in the TTT program are designed to follow a pilot-copilot principle, meaning that young or inexperienced tutors are closely supervised by more experienced tutors or even senior tutors who are tasked with supervising. Therefore, new tutors are always accompanied by a senior tutor during their first US course. During these courses, the senior tutor will stay within reach in the background and only engage when needed. The new tutors receive feedback after every course day during their first course.

**Appendix S2**

***Pretest example***

Name

Standard Plane (8 Points)

Draw and label the section plane labeled 'Epigastric sagittal plane, left paramedian: aorta'. Include the following specified standard values: reference luminal diameter of the abdominal aorta, measured cranially and caudally to the level of the renal arteries.

Theoretical Questions (2Points)

Please note: If you provide more answers than requested, only the first answer listed will be considered

1. Name two of the just drawn structures visible in the epigastric sagittal standard plane that could be easily confused with lymph nodes.
2. Name the two types of aortic dilatation. Please list the cutoffs (diameter) used to define these.

**Appendix S3**

***OSCE rotation evaluation form example***

Name

Practical Part (20 Points)

**Assignment**

A patient with known aortic ectasia comes to your practice for a check-up. Visualize the aorta in a sagittal plane in the upper abdomen. Measure its diameter supra- and infrarenally and compare it with the learned standard values. Then, identify all the vascular structures on the recorded ultrasound image that you would expect on the corresponding standard plane of the retroperitoneum.

**Checking the transducer orientation** (1 point)

**Explanation of the procedure**  (2 points)

**Instructing patient’s breathing and asking them to maintain breath** (2 points)

**Positioning of the transducer** (2 points)

1 point for positioning, 1 point for accurate naming of the standard plane

**Continuous scan through the aorta**  (2 points)

One complete scan in the upper abdomen is sufficient

**Visualization of the aorta in the sagittal plane** (2 points)

Suboptimal visualization of the vessel outlets after complete scan: 1 point

**Measuring the aortic luminal diameter**

**Suprarenal measurement**

- Correct position cranial to the celiac trunk and (1 point)

accurate measurement perpendicular to the wall

- Comparison with standard value (< 2.5 cm) (1 point)

**Infrarenal measurement**

- Correct position caudal to the Superior mesenteric artery (1 point)

and accurate measurement perpendicular to the wall

- Comparison with standard value (< 2.0 cm) (1 point)

**Indentification of vascular structures in the retroperitoneum**  (5 points)

**Structures to be identified**

- Aorta
- Celiac trunk
- Superior mesenteric artery
- Left renal vein
- Portal confluence or portal vein or splenic vein

Theoretical Part (10 Points)

**Name two typical characteristics of the aorta compared to** (2 points)

**the inferior vena cava.**

**Possible Answers**

- Thicker wall
- Single pulse
- Non-compressible
- Absence of erythrocyte swarms
- Lumen appears more hyperechoic

**What diameters of the aorta are considered pathological?**

- Aortic ectasia: 2.5 - 3cm (1 points)
- Aortic aneurysm: > 3cm (1 points)

**Name four signs of increased risk of aortic rupture.** (4 points)

**Possible Answers**

- Progressive dilatation
- Diverticular (saccular) shape
- Diameter exceeding 5cm
- Detected dissection
- Eccentric lumen

**Identify common causes of aortic aneurysm.** (2 points, *0.5 point per answer*)

**Possible Answers**

- Arteriosclerosis
- Traumatic damage
- Congenital vessel wall weakness
- Infections

**Appendix S4**

***Statistical Item Analysis of MCQs***

**Appendix S5**

***MCQ Examples***

Example Question 1

The caudate lobe of the liver typically borders:

a) The inferior vena cava

b) The filled gallbladder

c) The right kidney

d) I don’t know

Example Question 2

Please examine the following ultrasound image and mark the incorrectly labeled structure.

1. Pancreatic Head
2. Aorta
3. Vertebra
4. I don’t know


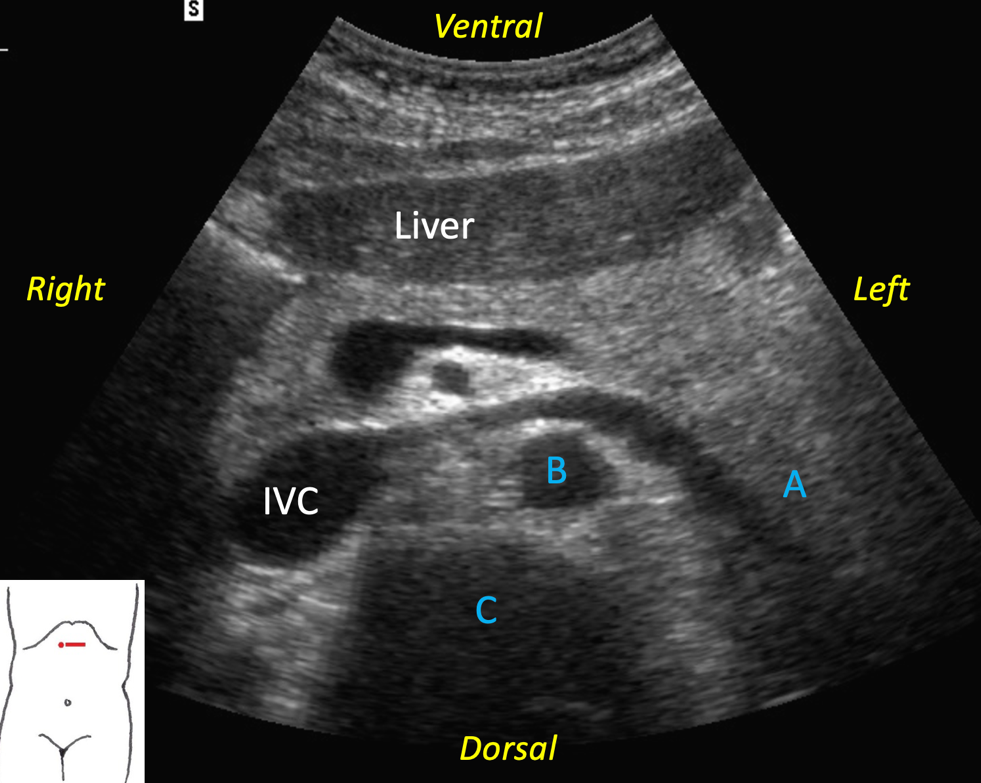


Example Question 3

Please examine the following MR image and identify the structure indicated by the arrow.

1. Celiac trunk
2. Superior mesenteric vein
3. Left renal vein
4. I don’t know


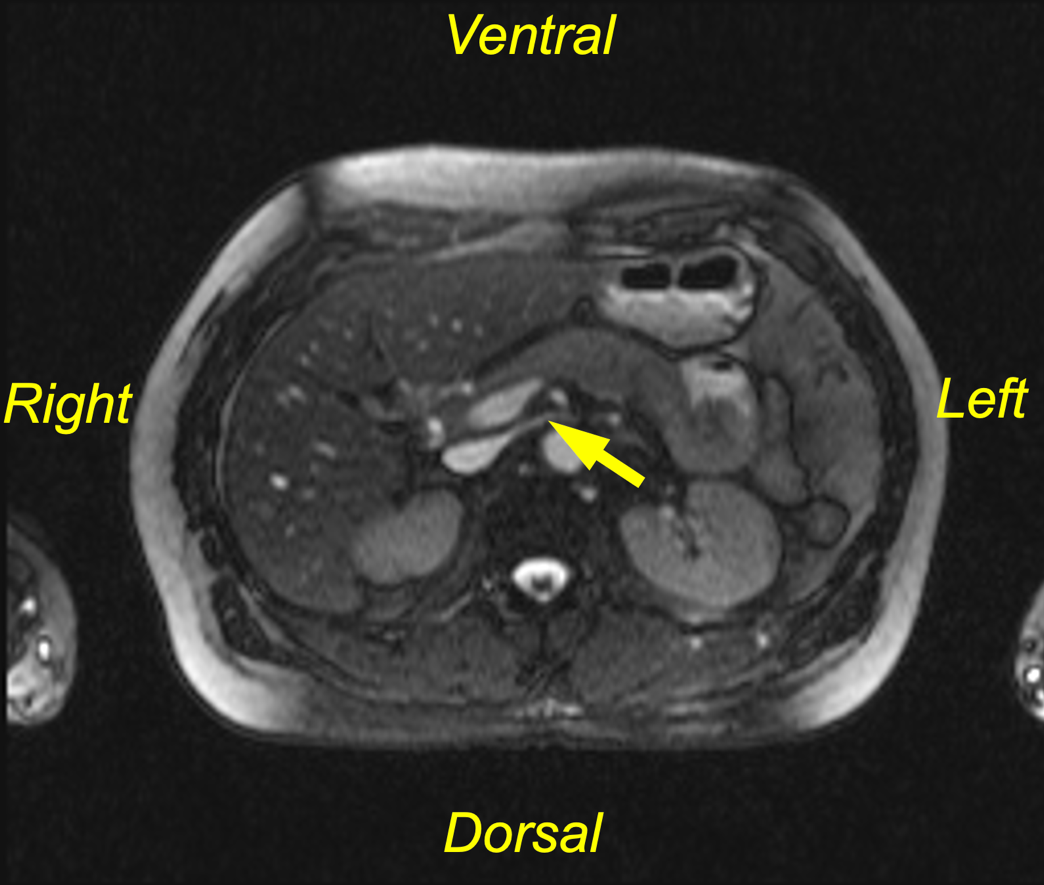

Supplement: Supplementary file 1 — Data S1: Supporting information. [file CA-39-541-s001.docx]
